# Transcriptome analysis and transcription factors responsive to drought stress in *Hibiscus cannabinus*

**DOI:** 10.7717/peerj.8470

**Published:** 2020-02-25

**Authors:** Xia An, Guanrong Jin, Xiahong Luo, Changli Chen, Wenlue Li, Guanlin Zhu

**Affiliations:** Zhejiang Xiaoshan Institute of Cotton & Bast Fiber Crops, Zhejiang Academy of Agricultural Sciences, Hangzhou, China

**Keywords:** Stress resistance, Kenaf, Transcription factor, Paired-end sequencing

## Abstract

Kenaf is an annual bast fiber crop. Drought stress influences the growth of kenaf stems and causes a marked decrease in fiber yield and quality. Research on the drought resistance of kenaf is therefore important, but limited information is available on the response mechanism of kenaf to drought stress. In this study, a transcriptome analysis of genes associated with the drought stress response in kenaf was performed. About 264,244,210 bp high-quality reads were obtained after strict quality inspection and data cleaning. Compared with the control group, 4,281 genes were differentially expressed in plants treated with drought stress for 7 d (the drought stress group). Compared with the control group, 605 genes showed differential expression in plants subjected to drought stress for 6 d and then watered for 1 d (the rewatering group). Compared with the rewatering group, 5,004 genes were differentially expressed in the drought stress group. In the comparisons between the drought stress and control groups, and between the drought stress and rewatering groups, the pathway that showed the most highly significant enrichment was plant hormone signal transduction. In the comparison between the rewatering and control groups, the pathways that showed the most highly significant enrichment were starch and sucrose metabolism. Eight transcription factors belonging to the AP2/ERF, MYB, NAC, and WRKY families (two transcription factors per family) detected in the leaf transcriptome were associated with the drought stress response. The identified transcription factors provide a basis for further investigation of the response mechanism of kenaf to drought stress.

## Introduction

Plant responses and adaptation to drought are complex (An et al., 2015; An et al., 2016; Liu et al., 2013; Nakashima et al., 2012; Shinozaki & Yamaguchi-Shinozaki, 2000). The plant activates a series of signal transduction mechanisms to resist drought stress (An et al., 2014; Hu et al., 2006). Signal transduction involves the expression of relevant genes and protein synthesis, which may result in changes to the antioxidant system and improvement in the plant’s resistance against drought stress (An et al., 2014; Li et al., 2016; Liu et al., 2013). Reactive oxygen species (ROS) are generated during photosynthesis and respiration, and the ROS content sharply increases under drought stress (An et al., 2014). Accumulation of ROS to a certain threshold in plant tissues results in degradation of the biological membrane system and consequently the cell ultrastructure is damaged (Sanchez et al., 2011). Drought tolerance is the product of combined action of a series of molecular, cellular, and physiological processes, including induction and inhibition of multiple genes, and enhanced antioxidant activity. Recent transcriptome analyses show that many genes respond to abiotic stresses in Arabidopsis (*Arabidopsis thaliana*) (Urano et al., 2009), rice (*Oryza sativa*) (Rabbani et al., 2003), soybean (*Glycine max*) (Su et al., 2014), wheat (*Triticum aestivum*) (Zhang et al., 2012) and ramie (*Boehmeria nivea*) (An et al., 2015). Researchers have studied in detail genes that play a pivotal role in the response to drought stress and the proteins encoded by these genes, including transcription factors. However, few such studies have been undertaken on kenaf (*Hibiscus cannabinus*)(Xu et al., 2013; Zhang et al., 2013).

Kenaf is an important raw material crop in the traditional textile industry. The processed products of kenaf fiber include automobile lining, agricultural paper films, fluff pulp, materials for sewage purification, soil conditioner, active carbon, and environment-friendly adsorption materials in addition to traditional products (e.g., hemp rope, hemp bag, carpet backing, canvas, and curtain cloth). Owing to its notable growth adaptability, especially its strong drought resistance, kenaf can be planted on mountain slopes and hilly topography, and thus does not compete for arable land with cereals (An et al., 2017). Therefore, kenaf is a crop that shows potential for wider cultivation to address the increasing demand for natural fibers.

Reflecting the paucity of genetic research on kenaf, only 20 expressed sequence tags for kenaf are registered in GenBank (as of August 28, 2018). In the present study, differential gene expression in kenaf leaves was compared under three treatments (daily watering for 7 d; drought stress for 7 d; and drought stress for 6 d and rewatering for 1 d) using an Illumina HiSeq™ 4000 high-throughput sequencing platform. Transcription factors that might participate in the drought resistance mechanism of kenaf were analyzed, thus laying a foundation for molecular breeding of kenaf for enhanced drought resistance.

## Material and Methods

### Preparation of plant materials and stress treatment

H368 shows the characteristics of high yield, drought resistance, and salt tolerance. H368 is suitable for planting in the Yangtze River, Huaihe River Basin, and South China regions. Seeds of ‘H368’ were donated by Professor Defang Li (Institute of Bast Fiber Crops, Chinese Academy of Agricultural Sciences, Changsha, China).

A pot culture experiment was performed. Each pot ( eight cm height, seven cm diameter) was filled with a soil mixture of the same weight (red soil: humus: vermiculite, 2:1:1, v/v/v). One kenaf plant was transplanted into each pot. All plants were cultivated in a greenhouse under white fluorescent lamps with a 16 h/8 h (light/dark) photoperiod and relative humidity of 65%–70%. When the plants had attained a height of about 30 cm, plants of uniform growth and morphology were selected as experimental materials. Three treatments were applied: in group C (the control group) plants were watered every day; in group D (the drought stress group) watering was withheld for 7 d; and in group R (the rewatering group) watering was withheld for 6 d and then plants were watered for 1 d. Leaves were collected, frozen in liquid nitrogen, and stored at −80 °C prior to analysis.

To ensure the quality of information analysis, we filtered the raw reads to obtain clean reads. The steps of data processing were as follows: (1) removal of the reads with adapters; (2) removal of reads that contained a proportion of N >10% (N indicates the base could not be determined); (3) removal of low-quality reads (the quality value Qphred ≤20 bases accounted for more than 50% of the total reads). Subsequent analysis was based on the clean reads. For study species in which a reference genome is not available, the clean reads must be spliced to obtain a reference sequence for subsequent analysis. In this study, we used Trinity to splice clean reads. Trinity is a highly efficient and stable transcriptome splicing software for RNA-seq data developed by the Broad Institute and the Hebrew University of Jerusalem. It combines three independent software modules to sequentially process a large number of RNA-seq data, namely Inchworm, Chrysalis, and Butterfly. We provided the assembled and annoteted sequence file as Supplementary Files 1 and 2.

### Determination of leaf relative water content and catalase activity

The relative water content (RWC) of the leaves was calculated in accordance with a previously reported method (An et al., 2014). Catalase (CAT) activity was determined following a previously described method (Nagamiya et al., 2007).

### RNA extraction and establishment of cDNA libraries

Total RNA was extracted from leaves of plants in the C, D, and R treatment groups using the RNAprep Pure Plant Kit (Tiangen Biotechnology, China), with two biological replicates per treatment group. The quality, concentration, and integrity of the RNA was analyzed using agarose gel electrophoresis and a NanoDrop™ 2000 spectrophotometer (Thermo Scientific, USA). An aliquot of 20 µg RNA from each of the six extracts was used for cDNA library construction. After the sample was tested, the mRNA was enriched with Oligo (dT) magnetic beads (Illumina, USA). Subsequently, fragmentation buffer (Invitrogen, USA) was used to break the mRNA into short fragments. Using mRNA as the template, single-strand cDNA was synthesized using random hexamers, and then double-stranded cDNA was synthesized by addition of buffer, dNTPs, DNA polymerase I, and RNase H (New England BioLabs, USA). The double-stranded cDNA was purified using AMPure XP beads (Beckman Coulter, USA). The purified double-stranded cDNA was first end-repaired, A-tailed, and ligated to the sequencing linker (T4 DNA polymerase, Klenow enzyme, and T4 polynucleotide kinase were purchased from the New England Labs), and the fragment size was selected using AMPure XP beads (Beckman Coulter, USA). Finally, PCR amplification was performed, and the PCR products were purified with AMPure XP beads (Beckman Coulter, USA) to obtain the cDNA library. Preliminary quantification of the library was performed using Qubit 2.0 (Thermo Fisher, USA), and the library was diluted to 1.5 ng/µl. The insert size of the library was detected using an Agilent 2100 Bioanalyzer (Agilent, USA). After confirmation of the expected insert size, the effective concentration of the library was determined by Q-PCR method. Accurate quantification (library effective concentration >2 nM) was performed to ensure library quality. The different libraries were pooled according to the effective concentration and the target data volume, and sequenced using paired-end reading of 150 bp (PE150) on an Illumina HiSeq 2500 platform.

### Sequence splicing and annotation

The cDNA libraries were subjected to high-throughput transcriptome sequencing. The Blastp tool was used to annotate all predicted protein coding sequences in the Non-redundant Protein database, GenBank, Swiss-Prot, and TrEMBL. Predicted proteins were first compared with information in the Swiss-Prot and TrEMBL databases using the following criteria: blastp and *E*-value <1e−5. The predicted proteins were annotated with gene ontology (GO) terms on the basis of a gene2go analysis using the GoPipe software (An et al., 2015). The main biochemical metabolic pathways and signal transduction pathways in which a specific protein participates can be determined by means of a pathway analysis (Kanehisa et al., 2010). Thus, the proteins were annotated with Kyoto Encyclopedia of Genes and Genomes (KEGG) pathway terms.

### Redundancy and enrichment analysis of differential expression

First, low-value sequences were removed to obtain clean reads, which were mapped onto spliced contigs. The quantities of reads from the two replicate samples for each contig were calculated, and were converted into reads per kilobase per million (RPKMs) (An et al., 2015; Mortazavi et al., 2008). Fragments Per Kilobase per Million mapped fragments (FPKM) refers to the number of fragments per kilobase length of a transcript in each million fragments, and was calculated using the formula FPKM=(1000000*C)/(N*L/1000), where FPKM is the expression level of transcript A, C is the number of reads aligned to transcript A, N is the total reads aligned to all transcripts, and L is the number of bases of transcript A. The MA-plot-based method with random sampling (MARS) model (Wang et al., 2010) in the DEGseq software package was used to calculate the expression abundance of each contig-represented gene in the two samples for each treatment group. Differentially expressed genes were identified as significant with a false discovery rate <0.001. After splicing and annotation, reads contained in the six samples (C1, C2, D1, D2, R1, and R2) were mapped to unigenes to calculate the RPKM value of each spliced unigene using the MARS model in the DEGseq software package. On this basis, differences in expression redundancy among the six samples were studied. A hypergeometric detection method was used for analysis of enriched GO terms/KEGG pathways among the differentially expressed genes (An et al., 2015).

### Identification of drought-responsive transcription factors

**Figure 1:**
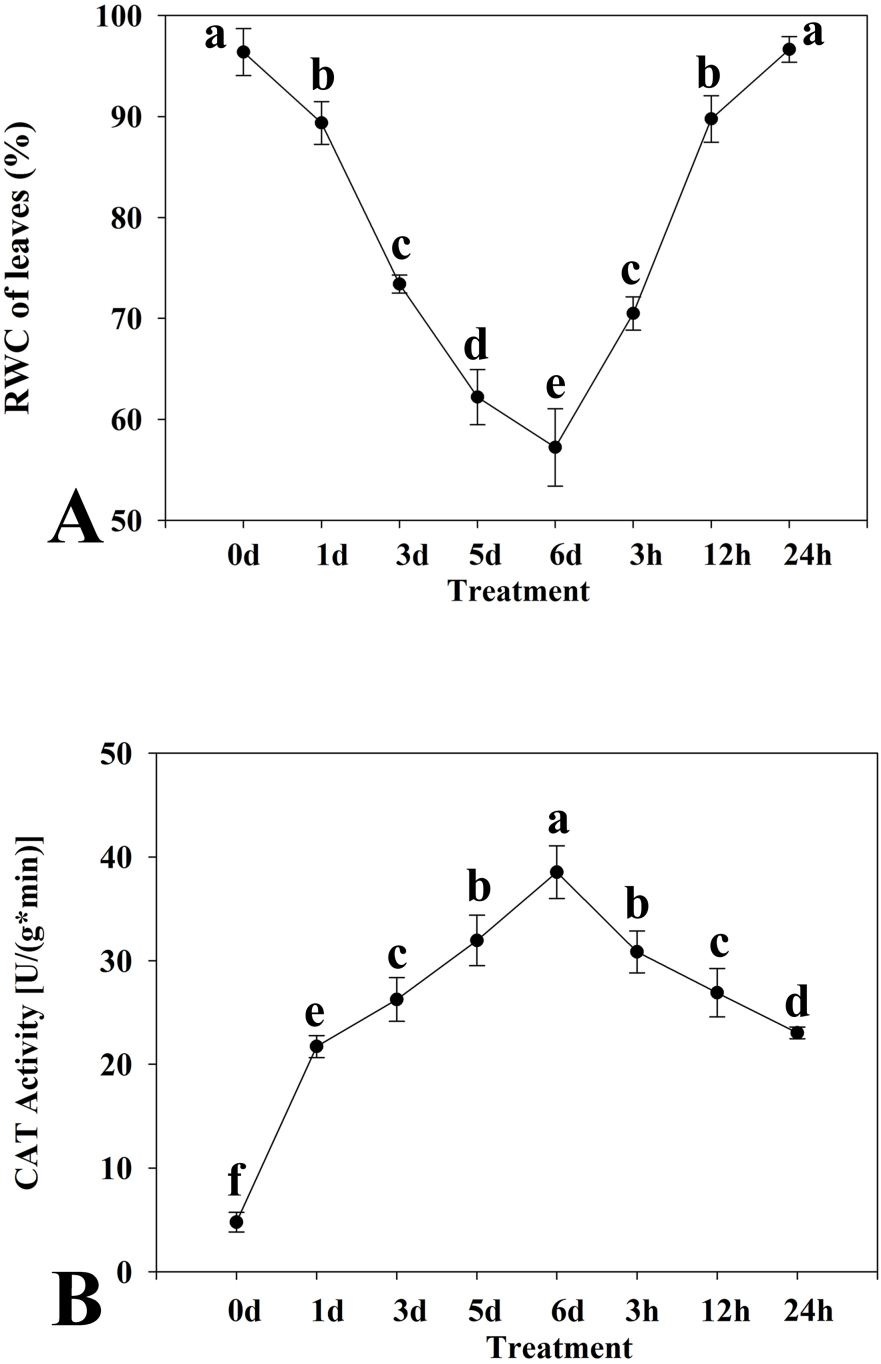
Relative water content (A) and catalase activity (B) in leaves of kenaf under drought stress. The leaves were harvested at 0, 1, 3, 5, and 6 days of drought stress treatment and at 3, 12, and 24 h after rewatering following drought stress for 6 d. Experiments were repeated three times. The values are mean ± standard deviation.

Among the differentially expressed unigenes belonging to four important families of transcription factors (AP2/ERF, MYB, NAC, and WRKY), those with expression patterns that coincided with the patterns of the physiological traits (as displayed in Fig. 1) were considered important. Primers of eight transcription factors was listed in Table 1.

**Table 1:** Primers of eight transcription factors. Cluster-5711.64302 is an internal reference gene.

NO.	Gene symbol	Forward primer(5-> 3)	Reverse primer(5-> 3)
1	Cluster-5711.64302	CTTCGCTCTTCCTTATTCGGTA	TGACCCATCCCAACCATAAC
2	Cluster-20186.19552	TTAAATCAAGTTCTGGTGCCG	TAGTTGCTGAGGCTCACTTC
3	Cluster-20186.58812	GGACTGGGCATTGTAACG	AAAGGACAGATGTAGGCATAGA
4	Cluster-20186.93998	AAGCCACCTTCTTCTGCATA	GACATTGTGGCATGATGATCC
5	Cluster-20186.44746	TCCTCAAGAAAGCGACGG	AATGGGACACCAGAAACAG
6	Cluster-20186.69423	ACAAGATAAGGTGTGTTCCG	CTGTACTAGCGACAGCCCA
7	Cluster-20186.22058	TTCGGCAGTGGCATAGAC	TCTCCATGTAAACAGTTCTGCT
8	Cluster-20186.19921	CGACGTGTTCAGTAACGG	ATGCTCGCTTTCTATGTACAAT
9	Cluster-20186.88151	TAGGCCAAGCATCAATGTTAAG	CCTAGAATTGGACGAATCGAC

## Results

### Physiological response of kenaf to drought stress

Two physiological indices of the kenaf response to drought stress, namely the RWC of leaves and CAT activity, were determined. The leaf RWC showed a trend to decrease in response to drought stress (Fig. 1A). The decline was especially distinct at 1 d after the onset of treatment. The leaf RWC was highest at 0 d of drought stress treatment and the minimum RWC was attained at 6 d. The RWC increased with rewatering after drought stress for 6 d. Thus, the critical time points detected were at 0 d and 6 d of drought stress and 24 h after rewatering. Activity of CAT initially increased and thereafter declined in response to drought stress (Fig. 1B). The CAT activity was highest at 6 d of drought stress and the lowest at 0 d.

Based on the critical time points at which RWC and CAT values changed under drought stress and rewatering treatment, three time points were selected (0 d and 6 d of drought stress treatment, and 24 h after rewatering treatment). Analysis of the transcriptome of the experimental materials under drought stress at these time points was conducted.

### RNA sequencing, reads splicing, and annotation

The RNA integrity number (RIN) is a measure of the integrity and degree of degradation of a RNA sample. The RIN value differs among samples and is typicall ≥6.3 (animals), 5.8 (plants and fungi), and 6 (pronuclear). In the present study, the RIN values of the six samples were 6.7, 7.5, 6.5, 7.4, 6.9, and 7.5. Following strict read quality inspection and data cleaning, a total of 264,244,210 high-quality reads were retained from 274,712,402 raw reads, which resulted in an approximate of 39.63 G clean bases (Table 2). Of these reads, 246,038 transcripts were obtained after splicing of the high-quality reads, of which 145,118 non-redundant unigenes were generated (Table 3). The sequence lengths for transcripts and unigenes ranged from 201 to 16,899 bp, whilst the unigenes possessed longer average sequence length (1,088 bp) than that of transcripts (759 bp, Table 4). The raw sequencing data are available at the NCBI database under the accession of PRJNA545389.

**Table 2:** Sequencing data quality statistics.

Sample	Raw reads	Clean reads	Clean bases (Gb)	Error (%)	Q20 (%)	Q30 (%)	GC (%)
C1	48343348	46583778	6.99 Gb	0.02	95.61	89.81	46.09
C2	41155916	39548104	5.93 Gb	0.03	94.69	88.07	45.60
D1	49584720	47815990	7.17 Gb	0.02	95.33	89.30	45.08
D2	46375520	44311540	6.65 Gb	0.02	96.18	90.57	45.13
R1	41845014	40359650	6.05 Gb	0.03	94.83	88.32	45.49
R2	47407884	45625148	6.84 Gb	0.02	95.28	89.14	45.86

**Notes.**

Raw reads: Statistics of the original sequence data, counting the number of sequencing sequences for each file in units of four behaviors.

Clean reads: The calculation method is the same as Raw Reads, Raw bases, and only the statistical files are filtered sequencing data. Subsequent bioinformatics analysis is based on Clean reads.

Clean bases: The number of sequencing sequences is multiplied by the length of the sequencing sequence and converted to Gb.

Error rate: Base error rate.

Q20, Q30: The percentage of bases whose Phred value is greater than 20 or 30 accounts for the total base.

GC content: The sum of the number of bases G and C as a percentage of the total number of bases.

**Table 3:** Splicing length frequency statistics table.

Transcript length interval	200–500 bp	500–1 kbp	1 k–2 kbp	> 2 kbp	Total
Number of transcripts	148,924	44,681	30,524	21,909	246,038
Number of genes	50,375	42,325	30,509	21,909	145,118

**Table 4:** Splicing length statistics. N50/N90 is defined as: Sorting the splicing transcripts from long to short according to the length, accumulating the length of the transcript, and the length of the spliced transcript not less than 50%/90% of the total length is N50/N90, which can be used to evaluate the splicing effect.

	Min length	Mean length	Max length	N50	N90	Total nucleotides
Transcripts	201	759	16,899	1,391	285	186,830,952
Genes	201	1,088	16,899	1,722	478	157,819,566

**Figure 2:**
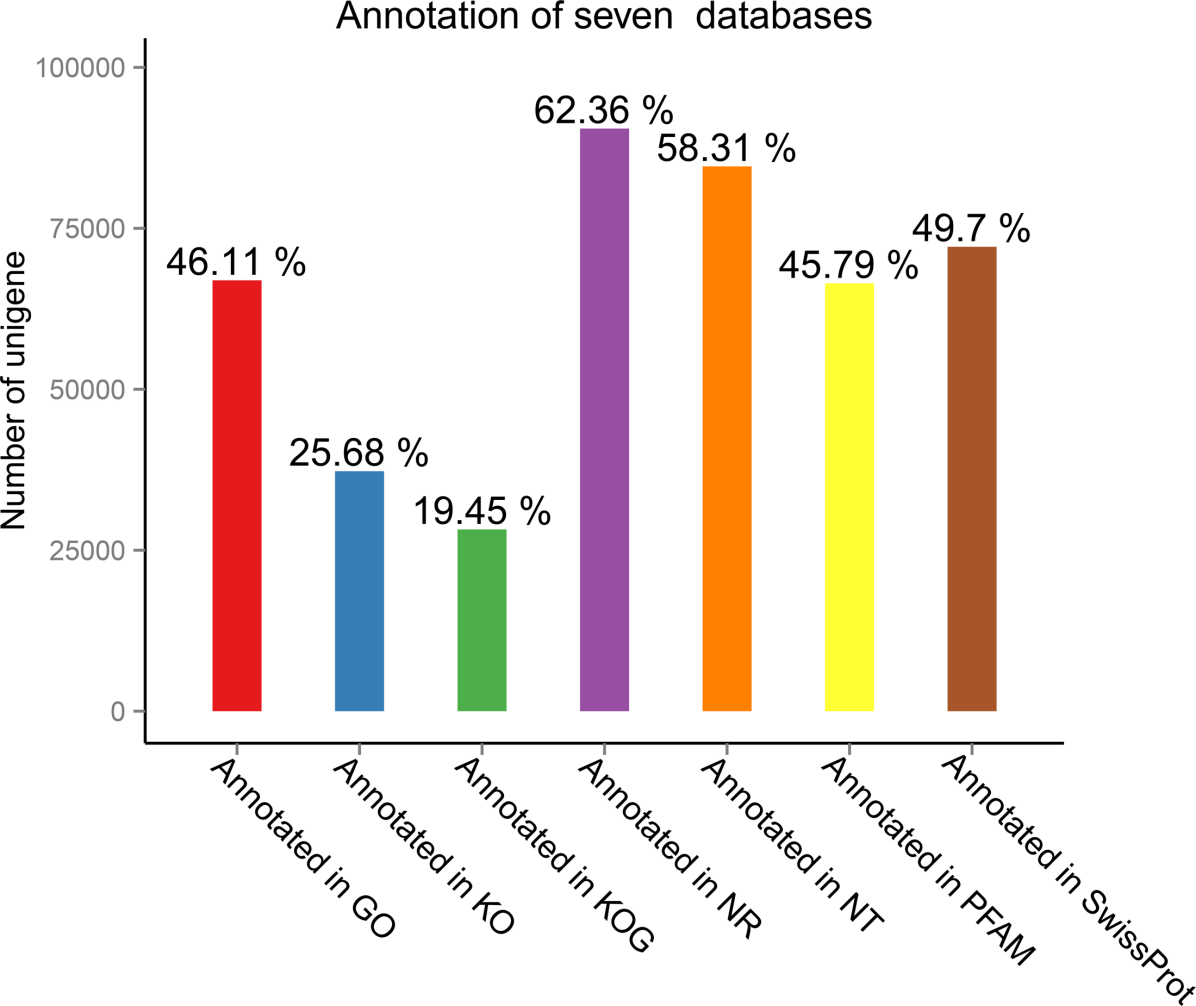
**Number of unigenes assignedto gene ontology (GO) term annotations**. The analysis was based on annotations in seven public databases (Nr, Nt, Swiss-Prot, KEGG [17], GO [21], COG, and Pfam).

Seven public databases, namely Nr, Nt, Swiss-Prot, KEGG (Kanehisa et al., 2010), GO (Young et al., 2010), COG, and Pfam, were used for functional annotation of the transcripts obtained through splicing. The numbers of unigenes assigned to GO term annotations are shown in Fig. 2.

### Functional classification and metabolic pathway distribution

**Figure 3:**
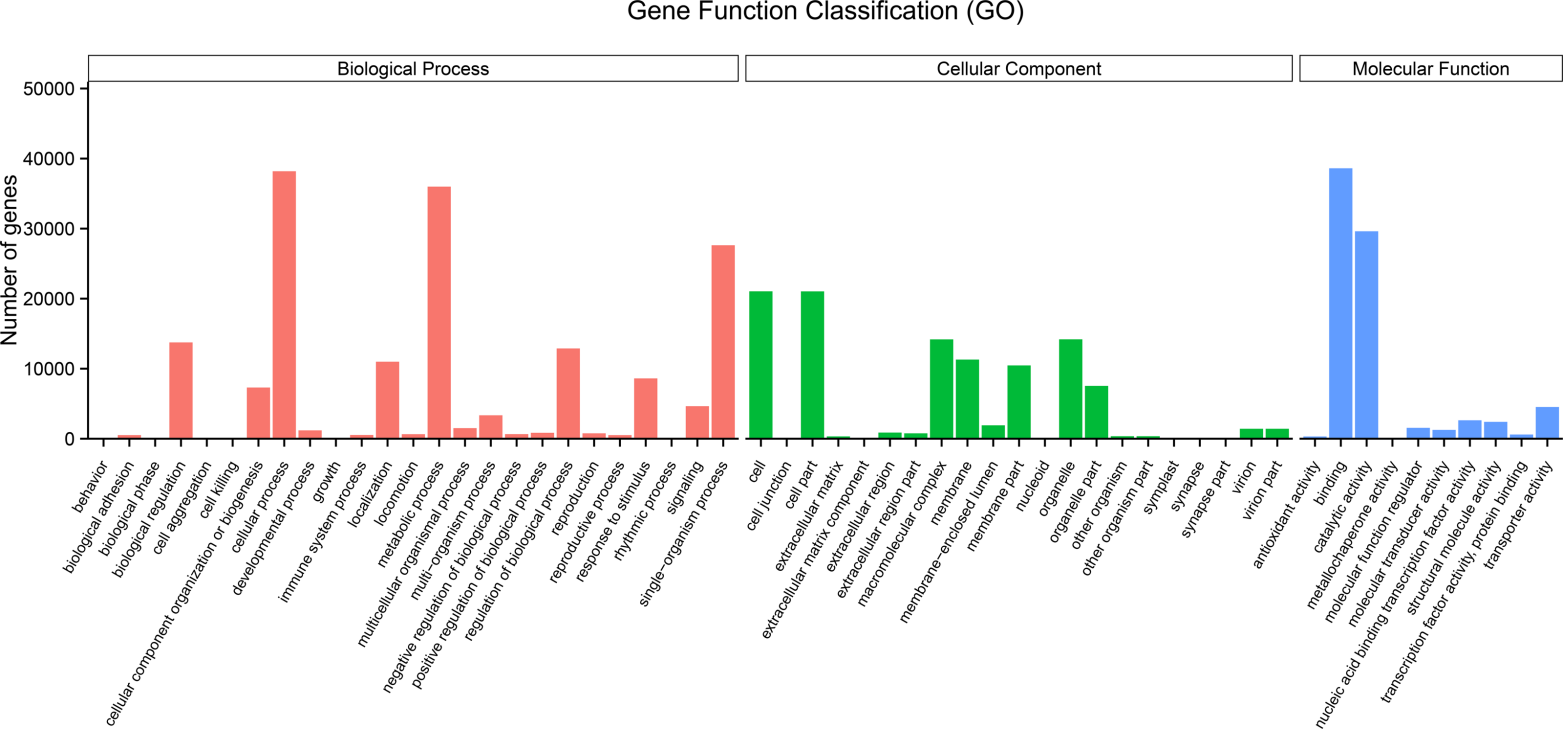
Histogram of gene ontology (GO) term classification. (A) Biological process (red); (B) cellular component (green); (C) molecular function (blue). The bars indicates the number of unigenes that were assigned GO annotations.

The GO terms are grouped into three categories, namely molecular function, cellular component, and biological process. An analysis of the unigenes was carried out by means of a Blastp similarity search of the GO database. The matched unigenes were classified into the three functional types as shown in Fig. 3 (Supplementary File 3). Among the biological process types, sequences were divided into 25 subtypes, of which the most frequently represented subtypes were “cellular process” and “metabolic process” (Fig. 3). Among the cellular component category, unigenes were divided into 21 subtypes, of which the most frequently represented subtypes were “cell” and “cell part” (Fig. 3). In the molecular function category, the matched unigene sequences were divided into 10 subtypes, of which the most frequent subtype was “binding” followed by “catalytic activity” (Fig. 3).

**Figure 4:**
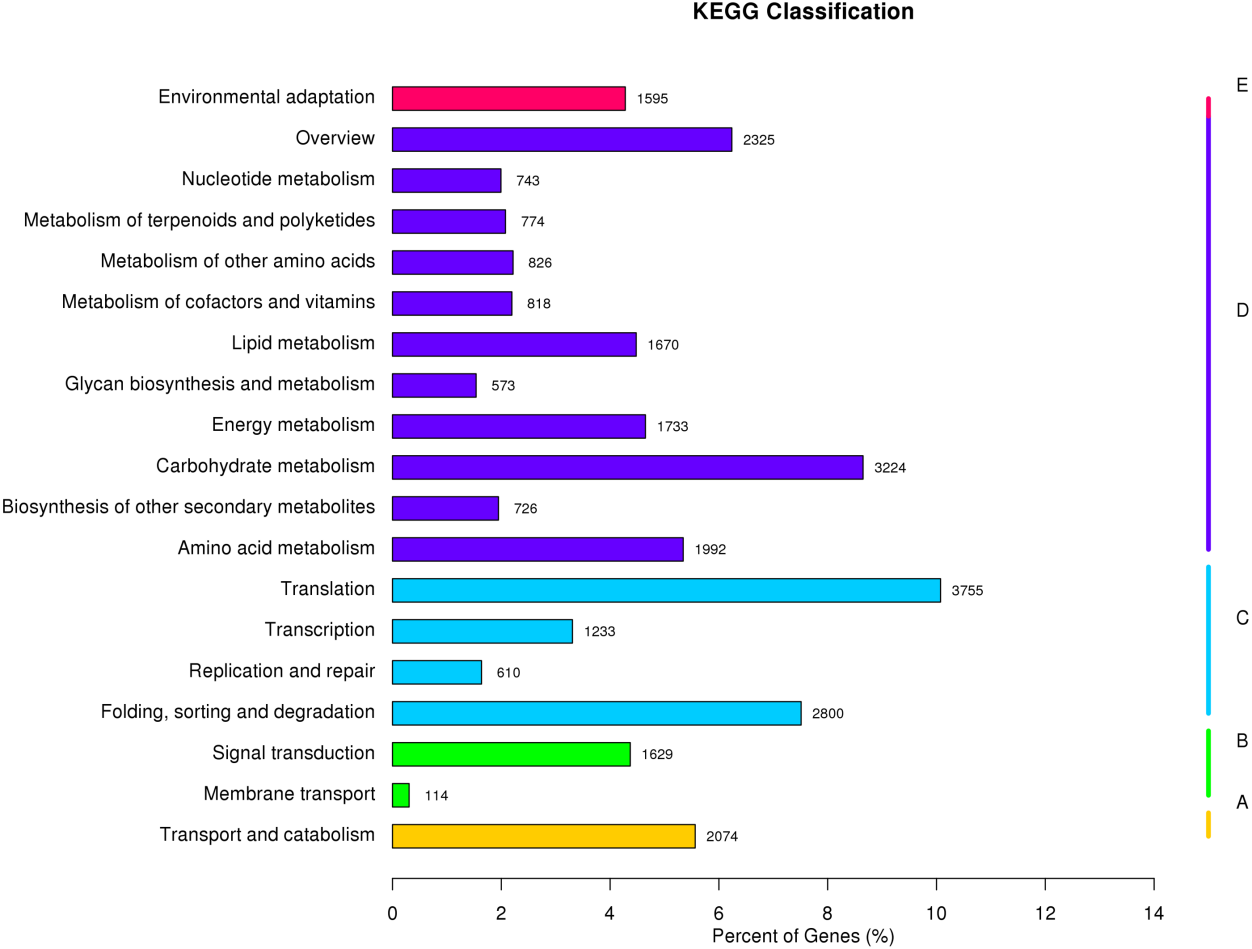
**KEGG classification of assembled unigenes**. KEGG metabolic pathways were assigned to five categories biochemical pathways: (A) cellular processes; (B) environmental information processing; (C) genetic information processing; (D) metabolism; and (E) organismal systems.

The KEGG Pathway database includes five major categories of pathways: Cellular Processes (A), Environmental Information Processing (B), Genetic Information Processing (C), Metabolism (D), and Organismal Systems (E). A network diagram showing the metabolic pathways enriched in the unigene data set is shown in Fig. 4 (File S4). Amino acid metabolism, biosynthesis of other secondary metabolites, carbohydrate metabolism, energy metabolism, glycan biosynthesis and metabolism, lipid metabolism, metabolism of cofactors and vitamins, metabolism of other amino acids, metabolism of terpenoids and polyketides were enriched. These pathways are associated with genetic information processing including translation, transcription, replication and repair, folding, sorting, and degradation. Metabolic pathways closely associated with cellular process and environmental information processing were shown in Fig. 4. These results provide a valuable resource for future investigation of metabolic pathways of kenaf.

### Screening of differentially expressed genes and cluster analysis of differential gene expression levels

**Figure 5:**
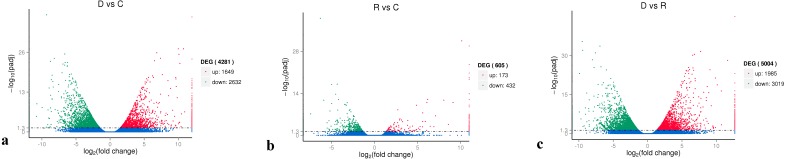
Analysis of differential gene expression between the treatment groups. (A) D vs. C; (B) R vs C; (C) D vs. R. The abscissa represents the fold change of gene expression in different samples; the ordinate represents the statistical significance of the change in gene expression. The smaller the corrected *p*-value, the larger the −log10 (corrected *p*-value), i.e., the difference was significant. Each data point in the figure represents an individual gene. The blue dots indicate genes with no significant differences in expression, the red dots indicate up-regulated genes with significant differences, and the green dots indicate down-regulated genes with significant differences.

**Figure 6:**
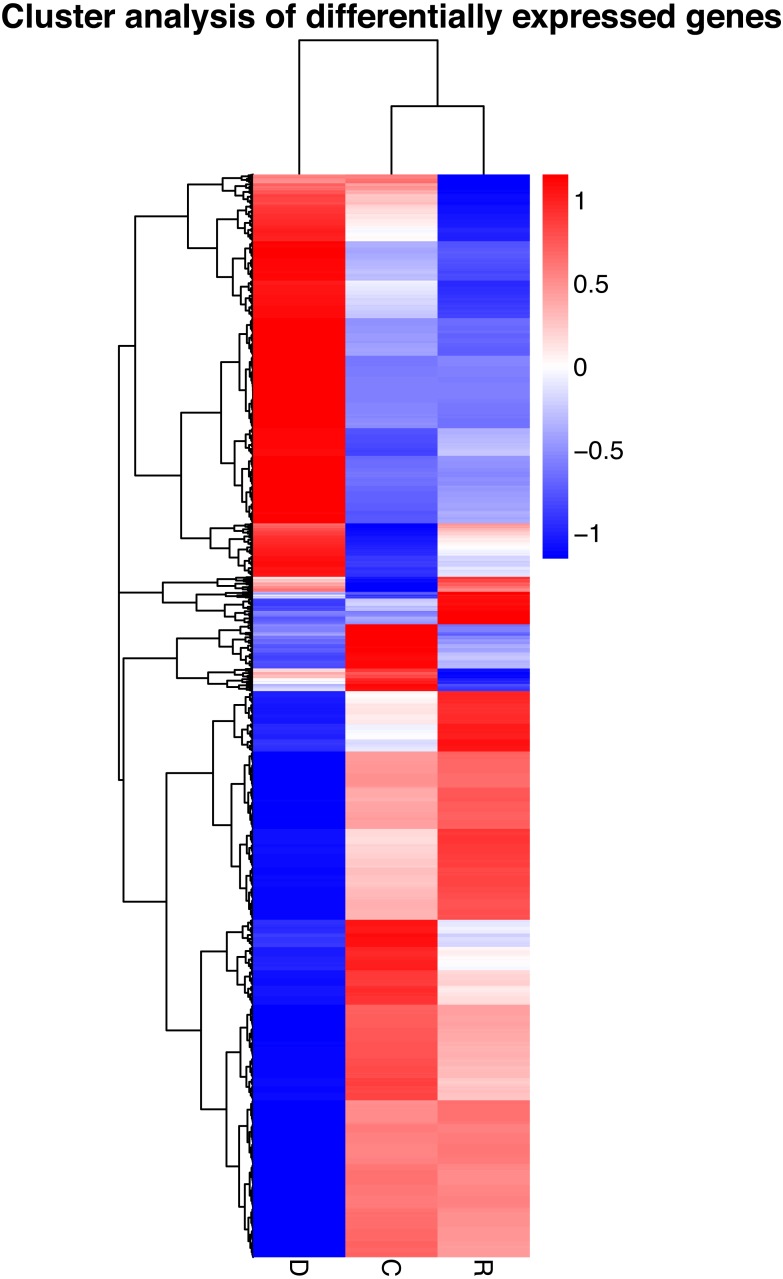
Hierarchical clustering map of differential gene expression in kenaf leaves under drought stress. Red indicates up-regulated genes and blue indicates down-regulated genes. The color ranges from red to blue, indicating that log10 (FPKM + 1) ranges from high to low. The *x*-axis represents the experimental conditions, and the *y*-axis represents the relative expression level.

On the basis of the RPKM method (Mortazavi et al., 2008), the MARS model (Anders & Huber, 2010) in the DEGseq software package was used to evaluate gene expression. The screening threshold value was *P* <0.05. Compared with group C, 4,281 genes were differentially expressed in group D, of which 1,649 genes were up-regulated and 2,632 genes were down-regulated (Fig. 5A). The reason that the number of down-regulated genes exceeded that of up-regulated genes after drought stress treatment may be associated with the decrease in leaf RWC. Compared with group C, 605 genes showed differential expression in group R, of which 173 genes were up-regulated and 432 genes were down-regulated (Fig. 5B). The number of down-regulated genes have exceeded that of up-regulated genes after rewatering treatment because drought stress may cause damage to plant leaf tissues and lead to insufficient ATP supply. Compared with group R, 5,004 genes were differentially expressed in group D, of which 1,985 genes were up-regulated and 3,019 genes were down-regulated (Fig. 5C). A cluster analysis of the differentially expressed genes was performed to assess clustering patterns of differential gene expression under the different treatments. A set of differentially expressed genes was obtained for each combination of treatment comparisons. The FPKM values of the combined set of differentially expressed genes present in all comparisons were selected for each treatment group (Fig. 6).

### KEGG analysis of differentially expressed genes

**Figure 7:**
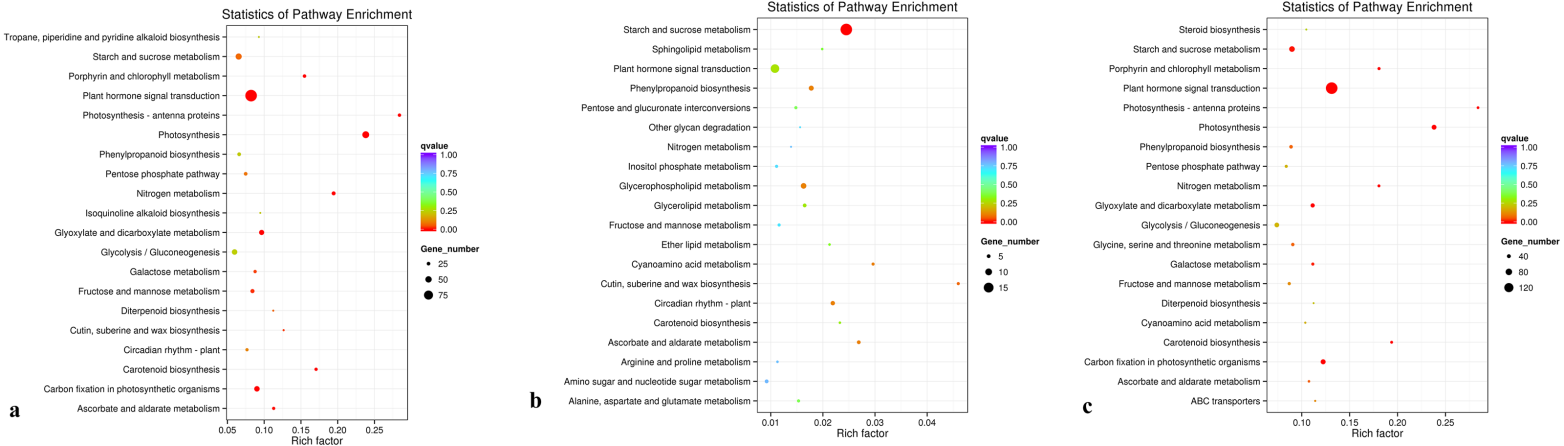
** KEGG pathwayenrichment scatter plot**. (A) Comparison of group D and C, (B) comparison of group R and C, and (C) comparison of group D with R.

The KEGG database was used to analyze gene products during the metabolic process. A KEGG enrichment scatter diagram for the differentially expressed genes provided a graphical presentation of the KEGG enrichment analysis. Comparisons between groups D and C, between groups R and C, and between groups D and R are presented (Figs. 7A, 7B, and 7C, respectively; Supplementary File 5). In Fig. 7, the degree of KEGG enrichment is indicated by the Rich factor, *q*-value, and number of genes enriched in a pathway. The Rich factor is the ratio of the quantity of genes belonging to the pathway among differentially expressed genes to the total number of genes belonging to the pathway among all annotated genes. The *q*-value is the *P*-value after multiple hypothesis testing and correction. The range of *q*-values is [0,1], and the closer the value is to 0, the more strongly significant the enrichment. Twenty pathways that showed the most highly significant enrichment were selected and are displayed in Fig. 7. For the comparisons between groups D and C, and between groups D and R, the pathway that showed the most highly significant enrichment was plant hormone signal transduction (Figs. 7A and 7C). For the comparison between groups R and C, the pathways showing the most highly significant enrichment were starch and sucrose metabolism (Fig. 7B).

### Determination of transcription factors responsive to drought stress

**Figure 8:**
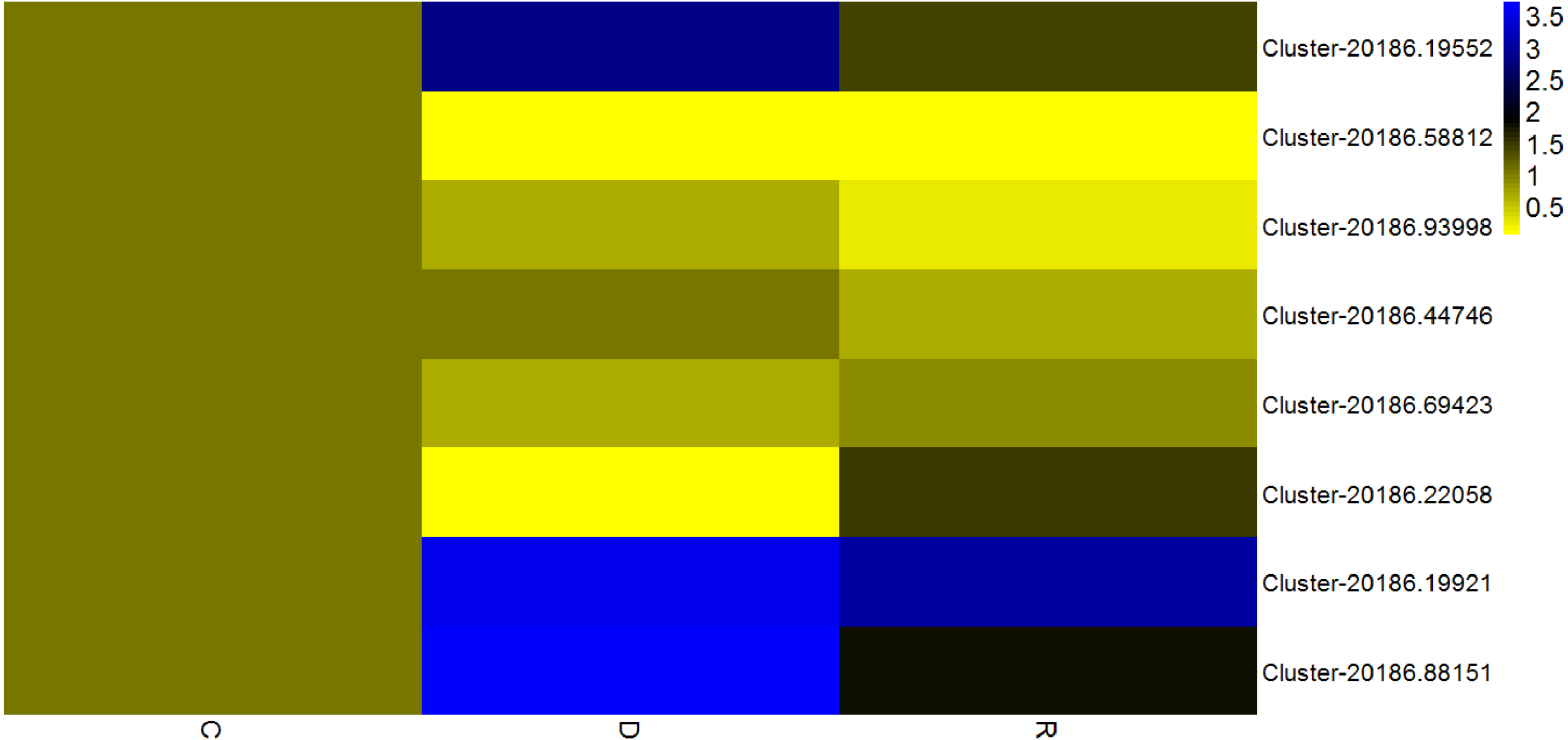
Expression analysis of eight transcription factorsby quantitative real-time PCR. According to the value of 2-ΔΔCT, the heat map in the diagram is obtained by taking the control group of each gene as 1. Cluster-5711.64302 is an internal reference gene.

Given that transcription factors are indicated to play an important role in the response to drought, identification of transcription factors responsive to drought stress in kenaf was an objective of the present study. Among differentially expressed unigenes, those that showed a consistent pattern of change in physiological properties (displayed in Fig. 1B) were deemed important. Therefore, unigenes from four transcription factor families (AP2/ERF, MYB, NAC, and WRKY) and that showed an “up-down” expression pattern in the leaves were deemed to show a consistent response with the physiological indices. Eight transcription factors with already known or assumed genetic coding were selected, of which two belonged to each of the AP2/ERF, MYB, NAC, and WRKY families. These transcription factors showed an up-down expression pattern under the influence of drought stress (Fig. 8).

## Discussion

### Transcription factor families associated with kenaf response to drought stress

Proteins that protect plant cells from damage caused by dehydration stress include osmo-regulatory proteins (Tamura et al., 2003), ionic channel proteins, transport proteins (Klein et al., 2004), and antioxidant or detoxification proteins (Bartels & Sunkar, 2005). The expression of these stress-related functional proteins is regulated by specific transcription factors to a large extent. Members of the AP2/ERF, MYB, NAC, and WRKY families have been verified have regulatory effects on defense and stress responses of other plant species (Hu et al., 2006; Klein et al., 2004; Zhu, 2002).

### AP2/ERF transcription factor family

The AP2/ERF family, which is a plant-specific transcription factor family, includes DRE connexin (DERBs), which can activate expression of genes that are responsive to abiotic stress and contain DRE/CRT (dehydration-responsive element/C-repeat) elements in their promoter (Licausi, Ohme-Takagi & Perata, 2013). In a previous study, 132 AP2/ERF transcription factors were analyzed in sesame (*Sesamum indicum*), of which the majority attained high expression levels in the roots and their main function was response to drought stress (Dossa et al., 2016; Licausi, Ohme-Takagi & Perata, 2013). In the present study, rewatering was implemented after drought stress treatment for 6 d; in response, two AP2/ERF transcription factors (Cluster-20186.19552 and Cluster-20186.58812) in kenaf leaves initially were up-regulated and thereafter were down-regulated. An up-down expression pattern might be because AP2/ERF transcription factors promoted expression of downstream functional genes associated with stress resistance, after expression of the AP2/ERF transcription factors was induced, so as to regulate diverse physiological and biochemical reactions in the plant. In this manner, the resistance of kenaf to drought stress was improved and the plant rapidly adapted to the stress condition and, as a result, the transcription factor expression level declined. Thus, in kenaf a certain feedback inhibition mechanism on gene expression products may operate. Drought stress-related transcription factors identified in the present study will enhance the understanding of gene expression, transcription regulation, and signal transduction in the response of plants to drought stress.

### MYB transcription factor family

It is considered that MYB transcription factors may exert important effects in the drought stress response, change expression levels of certain drought-related genes, and influence physiological reactions so as to overcome the adverse condition (Zhang et al., 2012). For example, AtMYB60 (a R2R3-MYB gene of Arabidopsis) is a transcription regulatory gene guarding physiological reactions of cells and participates in the regulation of stomatal movement (Cominelli et al., 2008). AtMYB60 also participates in the resistance of plants to drought stress (Cominelli et al., 2005). In kenaf leaves under the drought stress condition in the present study, MYB transcription factors (Cluster-20186.93998 and Cluster-20186.44746) initially were up-regulated and thereafter were down-regulated following rewatering after drought stress treatment. The MYB transcription factors might exert important effects in the regulation of stomatal movement and water-retention of kenaf leaves. When the stomata were closed and water loss was reduced, the resistance of kenaf to drought stress would be improved and represent adaptation to the stress environment and, as a result, the gene expression level was reduced. In addition, reduction in the severity of drought stress after rewatering might result in the decline in expression level. However, this hypothesis requires verification in further experiments.

### NAC transcription factor family

The plant-specific NAM, ATAF1-2, and CUC2 (NAC) family constitutes one of the largest transcription factor families (Bu et al., 2008; Olsen et al., 2005). NAC transcription factors participate in regulation of plant growth and development as well as control and defense of plant hormones (Tran et al., 2004). NAC family members participate in the plant response to abiotic stress, which can directly or indirectly regulate expression of responsive genes under drought and high-salinity stress (Hu et al., 2006). Participation of NAC transcription factors in regulation of the response to drought stress was first reported in Arabidopsis. Subsequently, NAC transcription factors have been shown to improve the drought resistance of rice. In the present study, NAC transcription factors (Cluster-20186.69423 and Cluster-20186.22058) showed an “up-down” expression pattern, which might be associated with the change in CAT activity associated with the drought stress response. Drought stress causes water deficit in plant tissues, influences metabolic activities, inhibits plant development, and reduces biological yield. As an important plant protection system, antioxidant enzymes play a crucial role in preventing excessive accumulation of ROS caused by stress conditions and exert a protective effect on cellular damage caused by lipid peroxidation. Thus, the greater the activity of protective enzymes, the stronger the plant resistance to stress.

### WRKY transcription factor family

WRKY transcription factors participate extensively in the plant response to abiotic stresses and play an important role in plant defense mechanisms. In rice, OsWRKY11 overexpression slows the rate of wilting of transgenic rice leaves, enlarges the area of photosynthetic tissues, reinforces drought resistance, and improves survival rate (Wu et al., 2009). In the present study, WRKY transcription factors (Cluster-20186.19921and Cluster-20186.88151) showed an “up-down” expression pattern, which might be associated with the change in CAT activity during the drought stress response. Wang et al. observed that the wheat *TaWRKY10* gene may be induced by multiple stresses, and was up-regulated under osmotic stress induced by polyethylene glycol treatment. Over-expression of *TaWRKY10* in transgenic tobacco conferred enhanced drought resistance, and contributed to a higher survival percentage of tobacco plants by regulating ROS scavenging, the osmotic balance, and expression of stress-associated genes (Wang et al., 2013).

## Conclusions

Reflecting the paucity of genetic research on kenaf, only 20 expressed sequence tags for kenaf are registered in GenBank (as of August 28, 2018). We have established a transcriptome analysis of genes associated with the drought stress response in kenaf and obtained about 264,244,210 bp high-quality reads. This transcriptome dataset will aid in understanding and carrying out future studies on the molecular basis of kenaf under drought stress.

##  Supplemental Information

10.7717/peerj.8470/supp-1 Supplemental Information 1 Assembled Transcriptome Unigene Part 1

10.7717/peerj.8470/supp-2 Supplemental Information 2 Assembled Transcriptome Unigene Part 2

10.7717/peerj.8470/supp-3 Supplemental Information 3 Annotated sequences

10.7717/peerj.8470/supp-4 Supplemental Information 4 The matched unigenes classified into three functional types

10.7717/peerj.8470/supp-5 Supplemental Information 5 Network diagram showing metabolic pathways enriched in the unigene dataset

10.7717/peerj.8470/supp-6 Supplemental Information 6 Comparisons of KEGG pathway enrichment between groups D and C, between groups R and C, and between groups D and R

## Additional Information and Declarations

### Data Availability

The following information was supplied regarding data availability: The raw sequencing data are available at NCBI: PRJNA545389.
